# Validation of a food frequency questionnaire for pregnant Swedish women in the NorthPop Birth Cohort Study against repeated 24-hour recalls

**DOI:** 10.1186/s12937-026-01357-z

**Published:** 2026-07-14

**Authors:** Richard Lundberg-Ulfsdotter, Maria Öström, Stina Bodén, Christina E. West, Magnus Domellöf, Elisabeth Stoltz Sjöström

**Affiliations:** 1 Department of Clinical Sciences, Pediatrics, Umeå University, Umeå, Sweden; 2 Department of Food, Nutrition and Culinary Science, Umeå University, Umeå, Sweden

**Keywords:** 24-hour dietary recall, Dietary assessment, Food frequency questionnaire, Food groups, NorthPop Birth Cohort Study, Nutrient intake, Pregnant women, Relative validity, Validation study

## Abstract

**Background:**

Diet during pregnancy significantly influences maternal and offspring health outcomes, including long-term risk of obesity, cardiovascular disease, asthma, allergic disease and diabetes. Food frequency questionnaires (FFQs) are cost-effective tools for dietary assessment but require validation to ensure accuracy. The objective of this study was to assess the validity of an FFQ developed for pregnant women in the NorthPop Birth Cohort Study (NorthPop) in Sweden.

**Methods:**

Pregnant women (*n* = 96) were recruited from NorthPop. Nutrient and food group intakes assessed by the FFQ were compared with intakes assessed by three 24-hour dietary recalls (24HR) using group mean comparisons, Spearman correlation coefficients, cross-classification, and Bland-Altman analysis.

**Results:**

The FFQ showed acceptable to very good correlations for most nutrients and food groups. Mean deattenuated correlation coefficients were 0.45 (0.15 to 0.73) for nutrients and 0.55 (0.32 to 0.80) for food groups. Energy adjustment altered individual nutrient correlations, but the overall mean remained unchanged at 0.45 (0.11 to 0.84); however, it reduced the mean correlation for food groups to 0.49 (0.17 to 0.70). Analyses of mean intake, cross-classification, and Bland-Altman plots showed acceptable agreement between the FFQ and the 24HR reference method.

**Conclusions:**

The FFQ evaluated in this study is a valid tool for assessing dietary intake at the group level among pregnant women in the NorthPop cohort. The results support the use of this FFQ to study relationships between diet and disease and to identify dietary factors during pregnancy.

**Supplementary Information:**

The online version contains supplementary material available at 10.1186/s12937-026-01357-z.

## Introduction

Pregnancy is a critical period during which maternal nutrition has a major influence on maternal and offspring long-term health [1]. Inadequate levels of key nutrients during crucial periods of fetal development may lead to reprogramming within fetal tissues, predisposing the infant to chronic conditions in later life [2]. Importantly, maternal nutrition during pregnancy may also influence the programming of the fetal immune system, thereby affecting the child’s risk of developing asthma and allergic disease [3]. Furthermore, intrauterine growth restriction followed by rapid catch-up growth in children may result in metabolic programming and increased risk of obesity, cardiovascular disease and diabetes in adulthood [4].

Increased fetal growth associated with maternal obesity and maternal gestational weight gain is also associated with an increased lifetime risk of obesity, cardiovascular disease, and diabetes in the offspring [5]. Maternal diet and gestational weight gain are also associated with maternal health issues and pregnancy complications [6, 7]. Deficiencies of key micronutrients are still common among pregnant women in Sweden [8, 9], and have considerable impact on long-term health outcomes in the offspring, not least neurodevelopmental problems in childhood and persistent mental health problems in adulthood [10]. To identify such deficiencies and address inadequate intakes, it is important to assess the dietary intake of pregnant women.

For this purpose, a food frequency questionnaire (FFQ) was developed for the dietary assessment of pregnant women in the NorthPop Birth Cohort Study (NorthPop), a population-based prospective cohort study conducted in northern Sweden with study design and data collection procedures previously described [11]. An FFQ is a retrospective method useful for estimating habitual food intake over extended periods. It is an appropriate tool for assessing dietary intake in various epidemiological settings, including studies of pregnant women [12]. Although FFQs are not suitable for accurately assessing true nutrient intakes at the individual level, they can effectively rank individuals by intake levels, distinguishing between low and high intakes [13]. FFQs have several other advantages, including ease of administration and cost-effectiveness [14]. As with all dietary assessment methods, the use of FFQs is subject to error and requires validation [15].

The objective of this study was to assess the validity of the FFQ developed for pregnant women in NorthPop. To determine the true validity of an FFQ would entail using a reference method that can monitor individual food intake over several months - a requirement rarely met in practice [16]. Therefore, relative validity is usually estimated by comparing FFQ data with data from an alternative dietary assessment method. Common reference methods for validating FFQs include 24-hour dietary recall (24HR), weighed food records, and comparison with biomarkers [17]. Since 24HR has a low risk of influencing the respondents’ dietary habits, has a low burden on the individual [15], and is cost-effective, 24HR was selected as the reference method for the present study.

## Methods

### Study participants and study design

Based on previous recommendations [13, 15], the target sample size was set to *n* = 115, with three 24HRs per participant. Participants were recruited from pregnant women enrolled in NorthPop. Inclusion criteria were pregnant women ≥ 18 years of age, comprehending the Swedish language, viable pregnancy at 14 to 24 weeks of gestation, and intention to give birth and reside in the catchment area in the next few years. As part of the NorthPop study protocol, participants received a two-part questionnaire with the first part administered at 34 weeks of gestation, followed by the second part one week later. The FFQ validated in this study is included in the second part.

A random sample of NorthPop participants at 27–28 weeks of gestation was contacted by telephone and informed of the validation study. Those who agreed to participate were mailed written information, a consent form, and a pre-stamped reply envelope. Upon return of the signed consent form, participants were enrolled in the validation study. The recruitment process was repeated eleven times between March 2021 and April 2022. To expedite recruitment, during the last three of the eleven recruitment rounds, all NorthPop participants at 27–28 weeks of gestation were invited to participate in the validation study, replacing the original approach of conducting random sampling. This study was reported according to the Strengthening the Reporting of Observational Studies in Epidemiology - nutritional epidemiology (STROBE-nut) guidelines [18] (Table S1).

### Dietary data collection

#### FFQ, the test instrument

The FFQ was administered as a web-based questionnaire to pregnant women participating in NorthPop and was adapted from the validated 84-item FFQ used in the Northern Sweden Health and Disease Study [19]. The FFQ used in NorthPop was an expanded and updated version including 125 food items tailored to capture contemporary Swedish dietary habits. In addition, the FFQ included six open-ended questions that allowed participants to report their intake of spreadable sandwich fats, sandwich toppings, vegetarian foods, condiments, vegetables, and beverages not specified among the predefined food items. All questions required a response before participants could proceed to the next question, i.e. omitting questions was not possible. FFQ questions were phrased as “How often do you eat...”, without specifying a reference period for participants to consider. The intake of nutritional supplements was not assessed.

Intake frequencies were assessed using two scales. Scale 1: Less often/never, 1–3 times/week, 4–6 times/week, 1–2 times/day and 3 or more times/day. Scale 2: Rarely/never, 1–3 items per week, 4–6 items per week, 1–2 items per day, 3–4 items per day, and 5 or more items per day. Scale 2 was used for slices (sliced toppings), cups (coffee and tea), and glasses (beverages). Scale 1 was used for all other items except for bread, fruits and berries, and milk. Bread intake was assessed by first asking how many slices the participant typically eats per day, then presenting six bread types for participants to rank by consumption frequency. Fruit and berry intake was assessed by first asking how often fruit and berries were consumed, then presenting ten fruit types and eight berry types for participants to mark those they typically consumed. Milk intake was assessed by first asking which of a list of five common types of milk the participant most often consumed (dairy and non-dairy milk), followed by a question on the average daily intake measured in deciliters.

For portion size assessment, the Swedish National Food Agency’s weight Table [20] was used. The weight table contains a list of approximately 1400 foods and dishes with corresponding weights for standard portions. A standard portion refers to the average portion consumed, based on data from weighed food records of adults in the Nordic countries. All weights in the weight table refer to the edible portion, excluding losses due to preparation and cooking.

The FFQ included questions about usual portion sizes for carbohydrate-dense foods (potatoes, rice, pasta, et cetera), protein-dense foods (fish, meat, eggs, soy products, et cetera), and vegetables. Each portion size was illustrated with photos showing 50%, 100%, 150%, and 200% of a standard portion, sourced from the Swedish National Food Agency’s weight table. The portion sizes of reported food intakes in these categories were adjusted accordingly.

The average daily intake of each food item was calculated by multiplying the daily frequency of consumption by the portion size. Average daily nutrient intakes were then calculated by multiplying the average daily food intake by the nutrient content of each food item according to version 20210503 of the Swedish Food Agency’s food database [21]. Missing values regarding nutrient content were assumed to represent zero content.

Data from the open-ended questions were analyzed using responses from the first 5000 completed FFQs in NorthPop. The most frequently reported foods in the open-ended questions across these 5000 FFQs were categorized into 16 additional food items. These items were then used to code responses to the open-ended questions in the current study. Calculation of average daily intake of foods and nutrients was then conducted using the same methods as for the predefined food items.

Finally, the food items in the FFQ were categorized into 13 food groups based on a classification system used by the Food and Agriculture Organization (FAO) [22], with modifications to better align with Swedish dietary habits (Table S2). Most food group names are intuitive, but “Discretionary foods” may need clarification. These foods are high in energy, added sugars, saturated fats, and/or salt, with minimal nutritional value. Examples include sweets and desserts, sugary beverages, and processed snacks.

#### 24HR, the reference method

**Fig. 1:**
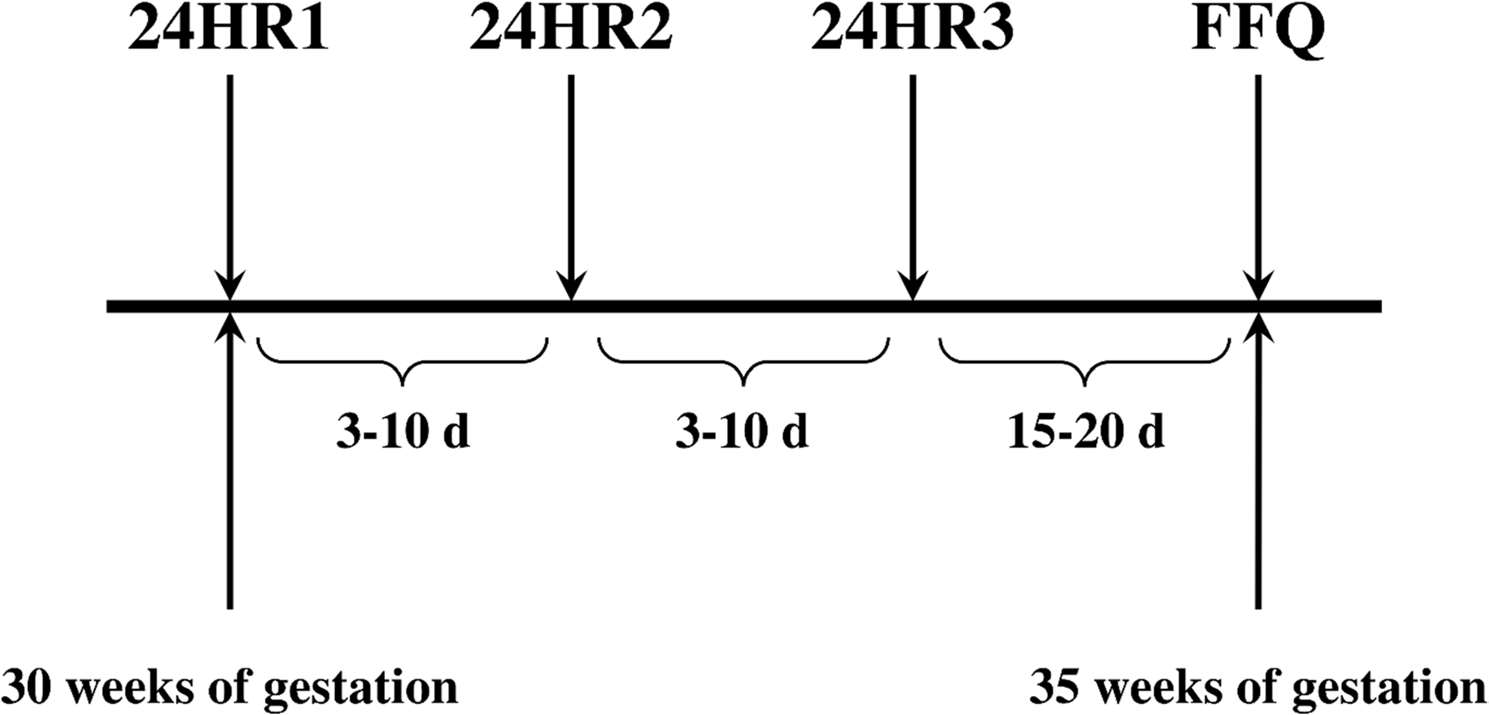
Timeline of the validation study for a food frequency questionnaire (FFQ) designed for pregnant Swedish women participating in the NorthPop Birth Cohort Study (2021–2022), showing the scheduling of the three included 24-hour dietary recalls (24HR1, 24HR2, 24HR3) and the FFQ

Three 24HRs were conducted by one out of six registered dietitians through telephone interviews on three nonconsecutive days between 30 and 33 weeks of gestation, capturing two weekdays and one weekend day (Fig. 1). During the interviews, participants were asked to recall all foods and drinks consumed during the preceding day. The 24HRs were conducted using a standardized interview guide and checklist developed within the research group, inspired by the Swedish Food Agency’s guide for administrating 24HR.

To facilitate participants’ portion size estimation, they received a portion guide published by the Swedish Food Agency [23] before the first 24HR. This guide contained photographs showing different portion sizes for various foods and beverages. Each portion size in the guide corresponded to a weight in grams, which was available in a separate document only for the dietitians. During the 24HRs, participants referred to the guide to report portion sizes consumed. Foods not available in the guide were estimated in household measurements or number of items. Participants were also prompted throughout each session to recall any forgotten foods. All data from the 24HRs were entered into the Nutrition Data software (Nutrition Data Sweden AB, Norsjö, Sweden) to generate nutrient intake information. The Nutrition Data software primarily uses nutrient content from the Swedish Food Agency’s food database, supplemented by their own database where foods are nutritionally calculated using ingredient lists and manufacturer information. Missing values regarding nutrient content were assumed to represent zero content.

The foods eaten according to the 24HRs were categorized into the same food groups as the FFQ food items. Foods reported in the 24HRs that did not belong to any specific food group were not included in the food group analysis. However, the nutrient analysis included all foods reported in the 24HRs.

### Background data

Data on parity, education, age at delivery, body mass index (BMI) measured at the first visit to the maternity care center, and weeks of gestation were obtained from the NorthPop-database.

### Statistical analyses

The validity of the FFQ was assessed using Spearman rank correlation between the FFQs and the averaged 24HRs. This approach was chosen because, even after log transformation, eleven of the 28 nutrients and 12 of the 13 food groups did not meet the assumptions of a normal distribution, as determined using the Shapiro-Wilk test. Implausible energy intake was assessed using cutoffs of < 500 kcal/day and > 5000 kcal/day, consistent with standard practice in recent large pregnancy cohorts [24–26]. No participants met these criteria. Intake of nutrients and food groups were energy adjusted using the residual method, which removes the influence of total energy while preserving the relative proportions [27]. After energy adjustments, 21 of the 28 nutrients and all 13 food groups were found to be outside the normal distribution assumptions.

To minimize attenuation of the correlation coefficient due to random day-to-day variation in the participants’ 24HRs, the correlation coefficients were deattenuated as recommended by Rosner and Willett [28]. The deattenuated correlation coefficient can be expressed as:

$$\:{r}_{t}\mathrm{=}{r}_{0}\sqrt{\left(1\mathrm{+}\frac{\lambda\:}{n}\right)}$$

where $\:{r}_{t}$ is the “true” deattenuated correlation coefficient, $\:{r}_{0}$ is the observed crude correlation coefficient, $\:\lambda\:$ is the ratio of intra- to interindividual coefficients of variation, and $\:n$ is the number of 24HRs per participant. A weak correlation was defined as *r* < 0.3, an acceptable correlation as 0.3 ≤ *r* < 0.5, a good correlation as 0.5 ≤ *r* < 0.7, and a very good correlation as *r* ≥ 0.7. These thresholds align with criteria used in previous systematic reviews and authoritative publications [13, 15, 17, 29, 30].

To assess the degree of misclassification between the FFQ and 24HR, the intake of nutrients and food groups were categorized into quintiles. Categorization into the same or adjacent quintiles was considered correct classification, and categorization into extreme quintiles was considered gross misclassification. Bland-Altman analyses [31] were performed to evaluate the agreement between nutrient and food group intake assessed using the FFQ and the 24HRs. Paired samples t-tests and Wilcoxon signed ranks tests were used to identify differences between means for parametric and non-parametric data, respectively. Comparisons of categorical variables were performed using Fisher’s exact test.

All analyses were performed using R version 4.4.2 (R Core Team, Vienna, Austria) and SPSS version 28.0.1.1 (IBM Corp., Armonk, NY, USA). Two-tailed *p* values < 0.05 were considered significant.

## Results

A total of 267 NorthPop participants were invited to the validation study. Of these, 115 provided written consent, and 94 completed all three 24HRs and the FFQ. Additionally, two participants who completed two 24HRs along with the FFQ were also included. The remaining 19 participants, who completed fewer than two 24HRs or did not complete the FFQ, were excluded, resulting in a final sample of 96 participants. No exclusions were made due to implausible energy intake.

Nutrient data were available for all nutrients across all food items in the FFQ, except for iodine, which was missing for dried fruit, light margarine, herbal tea, fruit yogurt, frozen vegetable mix, white rice, porridge, marmalade, and vinaigrette. Among the 1261 unique food items reported in the 24HRs, nutrient data were available for all nutrients except for iodine (missing for 206 items), cholesterol (missing for nine items), and beta-carotene (missing for three items).

Table 1 displays a comparison between the characteristics of participants in the validation study and those of participants in NorthPop (*n* = 5650). The only significant difference between the groups was observed regarding parity, where a smaller proportion of women in the validation study had two or more previous pregnancies (8.3%) compared to the NorthPop participants (15.9%).

**Table 1:** Characteristics of the participants included in the validation study and of a sample of participants in the NorthPop Birth Cohort Study (NorthPop)

	Validation study (*n* = 96)	NorthPop^a^ (*n* = 5650)
	Mean ± SD	Mean ± SD
Age at delivery, years	31.0 ± 4.4	30.9 ± 4.4
Body Mass Index^b^, kg/m²	25.5 ± 5.3	25.0 ± 4.7
Timepoint for 24HR1, weeks of gestation	30.4 ± 0.8	
Timepoint for 24HR2, weeks of gestation	31.7 ± 0.5	
Timepoint for 24HR3, weeks of gestation	33.0 ± 0.7	
Timepoint for FFQ, weeks of gestation	35.4 ± 0.6	35.5 ± 0.7
Parity	**Percent**	**Percent**
0	50.0	46.9
1	41.7	37.2
2 or more	8.3	15.9*
Highest educational level		
University	69.8	69.9
High school	27.1	26.5
≤Elementary school	3.1	3.6

*24HR 1-3* 24-hour dietary recall 1st, 2nd and 3rd recall, *FFQ* Food frequency questionnaire

^a^ NorthPop participants enrolled at the start of the validation study, with all background data available

^b^ Measured at the first visit to the maternity care center, usually at 7–12 weeks of gestation

* *p* < 0.05

Table 2 presents the mean daily energy and nutrient intakes assessed using the FFQ and 24HRs, along with the FFQ-derived mean intakes expressed as percentage of the 24HR-derived mean intakes. There were no significant differences in mean intakes between the FFQ and the 24HRs for 25% of the nutrients. The FFQ overestimated intakes of protein, carbohydrate, and most of the B vitamins and minerals. Conversely, intakes of fat, most of the fat-soluble nutrients, and calcium were underestimated with calcium being the only underestimated mineral.

**Table 2:** Mean values with standard deviations (SD) for food frequency questionnaire (FFQ) and 24-hour dietary recall (24HR) assessment of daily energy and nutrient intake, and FFQ as percentage of 24HR in 96 pregnant women

Nutrient	Mean FFQ ± SD	Mean 24HR ± SD	FFQ % of 24HR
Energy, kcal	1920 ± 601	1977 ± 427	97
Protein, g	86.7 ± 32.3	75.4 ± 18.7	115^*^
Fat, g	62.7 ± 21.9	86.0 ± 23.4	73^*^
Carbohydrates, g	239 ± 79.5	215 ± 52.1	111^*^
Fiber, g	24.0 ± 12.1	20.1 ± 6.6	120^*^
Total saturated fatty acids, g	24.0 ± 9.7	34.8 ± 10.9	69^*^
Total monounsaturated fatty acids, g	23.7 ± 8.2	32.8 ± 9.2	72^*^
Total polyunsaturated fatty acids, g	9.8 ± 4.1	11.9 ± 4.1	83^*^
Cholesterol, mg	262 ± 118	272 ± 122	96
Retinol, RE	460 ± 238	822 ± 475	56^*^
Beta-carotene, µg	1904 ± 1974	2921 ± 2481	65^*^
Vitamin D, µg	8.0 ± 3.2	8.4 ± 3.3	95
Vitamin E, mg	10.0 ± 3.6	12.5 ± 4.7	80^*^
Thiamine, mg	1.4 ± 0.50	1.2 ± 0.44	112^*^
Riboflavin, mg	1.6 ± 0.64	1.6 ± 0.50	101
Vitamin C, mg	95.1 ± 53.8	103 ± 69.1	92
Niacin equivalents, NE	37.5 ± 13.9	30.6 ± 9.5	123^*^
Vitamin B6, mg	1.7 ± 0.64	1.5 ± 0.50	116^*^
Vitamin B12, µg	4.9 ± 2.4	4.4 ± 2.1	111^*^
Folate, µg	285 ± 120	276 ± 88.2	103
Iodine, µg	225 ± 105	188 ± 63.8	120^*^
Phosphorus, mg	1516 ± 533	1383 ± 359	110^*^
Iron, mg	11.1 ± 6.2	8.3 ± 3.1	133^*^
Calcium, mg	910 ± 393	986 ± 347	92^*^
Potassium, mg	2951 ± 1013	2696 ± 647	109^*^
Magnesium, mg	349 ± 132	290 ± 77.9	120^*^
Selenium, µg	39.0 ± 15.0	37.6 ± 14.7	104
Zinc, mg	12.8 ± 6.5	10.1 ± 2.7	126^*^

Paired Wilcoxon signed rank test

^*^ *p* < 0.05

Spearman correlation coefficients for nutrient intakes assessed using the FFQ and the 24HRs are presented in Table 3. Without energy adjustment, 22 of the 28 nutrients had acceptable (*r* ≥ 0.3) to very good (*r* ≥ 0.7) deattenuated correlations. Five nutrients had weak (*r* < 0.3) correlations, and one had a non-significant correlation. After excluding energy, 22 of the 27 nutrients had acceptable to very good correlations following energy adjustment, although the distribution within these ranges changed. Fourteen nutrients showed increased correlations, 12 nutrients showed decreases, and the correlation for one nutrient was no longer statistically significant. Notable changes included fiber (good to very good), calcium (very good to good), and niacin equivalents (weak to good). The correlation for retinol decreased the most, from good to weak. Iodine was not statistically significant after energy adjustment, and thiamine remained non-significant. Deattenuated values consistently showed stronger correlations than crude values. Both the mean deattenuated correlation and the mean deattenuated energy adjusted correlation were 0.45.

**Table 3:** Spearman correlation coefficients comparing daily nutrient and energy intake estimates from food frequency questionnaires and 24-hour dietary recalls in 96 pregnant women

Nutrient	Crude	Deattenuation factor	Deattenuated	Energy adjusted and deattenuated
Energy	0.39^*^	1.19	0.47*	
Protein	0.29^*^	1.17	0.34*	0.44*
Fat	0.33^*^	1.23	0.40*	0.41*
Carbohydrates	0.42^*^	1.22	0.52*	0.41*
Fiber	0.56^*^	1.18	0.66*	0.84*
Saturated fatty acids	0.38^*^	1.20	0.45*	0.41*
Monounsaturated fatty acids	0.28^*^	1.29	0.36*	0.41*
Polyunsaturated fatty acids	0.34^*^	1.23	0.42*	0.51*
Cholesterol	0.43^*^	1.20	0.51*	0.66*
Retinol	0.46^*^	1.12	0.51*	0.24*
Beta-carotene	0.42^*^	1.24	0.52*	0.35*
Vitamin D	0.51^*^	1.23	0.62*	0.49*
Vitamin E	0.32^*^	1.16	0.38*	0.41*
Thiamine	0.12	1.20	0.15	0.11
Riboflavin	0.50^*^	1.18	0.59*	0.64*
Vitamin C	0.46^*^	1.20	0.55*	0.50*
Niacin equivalents	0.25^*^	1.18	0.29*	0.51*
Vitamin B6	0.23^*^	1.18	0.27*	0.24*
Vitamin B12	0.52^*^	1.13	0.58*	0.57*
Folate	0.44^*^	1.18	0.53*	0.54*
Iodine	0.22^*^	1.27	0.28*	0.22
Phosphorus	0.49^*^	1.14	0.56*	0.54*
Iron	0.26^*^	1.28	0.33*	0.32*
Calcium	0.63^*^	1.16	0.73*	0.55*
Potassium	0.37^*^	1.18	0.43*	0.45*
Magnesium	0.46^*^	1.16	0.54*	0.65*
Selenium	0.23^*^	1.26	0.29*	0.32*
Zinc	0.23^*^	1.24	0.28*	0.29*

A weak correlation was defined as *r* < 0.3, an acceptable correlation as 0.3 ≤ *r* < 0.5, a good correlation as 0.5 ≤ *r* < 0.7, and a very good correlation as *r* ≥ 0.7

^*^ *p* < 0.05

Table 4 presents the cross-classification of reported nutrient intakes into quintiles, comparing estimates from the FFQ with the mean of the 24HRs. Without energy adjustment, most of the FFQ estimates were correctly classified, with rates ranging from 53% for iodine to 77% for fiber. Following energy adjustment, 11 nutrients showed increases in correct classification, 15 nutrients showed decreases, and two nutrients did not change. Notable changes were obtained for fiber, niacin equivalents, and iodine, which increased from 77% to 84%, 55% to 66%, and 53% to 60% respectively, and for calcium, which decreased from 76% to 65%. The mean proportion of correctly classified FFQ estimates was 66% across all nutrients regardless of energy adjustment. Gross misclassification rates without energy adjustment ranged from 0% for calcium to 7% for iron. Following energy adjustment, gross misclassification rates ranged from 0% for fiber, vitamin B12 and calcium to 8% for iodine. The mean misclassification rate was 3% regardless of energy adjustment

**Table 4:** Cross-classification of reported energy and nutrient intakes into quintiles, comparing the food frequency questionnaire with the 24-hour dietary recalls in 96 pregnant women

Nutrient	Crude		Energy adjusted		
	Correct classification (%)^a^	Gross misclassification (%)^b^	Correct classification (%)^a^	Gross misclassification (%)^b^	
Energy	66	1	65		1
Protein	61	3	64		2
Fat	60	6	64		4
Carbohydrates	67	2	65		1
Fiber	77	2	84		0
Saturated fatty acids	62	4	62		5
Monounsaturated fatty acids	68	5	67		6
Polyunsaturated fatty acids	67	6	67		2
Cholesterol	70	4	75		3
Retinol	69	2	65		5
Beta-carotene	65	2	61		4
Vitamin D	73	2	71		3
Vitamin E	67	3	65		3
Thiamine	54	3	50		6
Riboflavin	74	2	74		1
Vitamin C	69	3	67		2
Niacin equivalents	55	4	66		2
Vitamin B6	58	5	54		4
Vitamin B12	72	2	70		0
Folate	65	2	69		3
Iodine	53	3	60		8
Phosphorus	68	1	67		2
Iron	65	7	66		5
Calcium	76	0	65		0
Potassium	68	3	64		3
Magnesium	71	4	72		1
Selenium	59	5	60		6
Zinc	54	4	62		3

^a^ Same or adjacent quintile

^b^ Extreme quintiles

The Bland-Altman plots showed consistent patterns across most nutrients, with relatively wide 95% limits of agreement (LoA) and a small proportion of participants outside the LoA (Supplementary Fig. 1). Most nutrients had no systematic differences between the two assessment methods. Ten of the 28 nutrients showed slight linear trends between the differences and means of the FFQ and 24HRs, as indicated by significant *p*-values for the regression slope. For five nutrients (energy, fiber, iron, magnesium, and zinc), lower intake levels tended to be underestimated, while higher intake levels tended to be overestimated by the FFQ compared to the 24HRs. In contrast, for another five nutrients (retinol, beta-carotene, vitamin E, thiamine, and vitamin C), lower intake levels were overestimated, and higher intake levels were underestimated. Some nutrients, for example fiber, retinol, vitamin C, niacin, and vitamin B12, showed a few extreme outliers in the Bland-Altman plots.

Table 5 presents the mean daily intakes of food groups assessed using the FFQ and 24HRs, along with the FFQ-derived mean intakes expressed as a percentage of the 24HR-derived mean intakes. Compared to 24HRs, the FFQ overestimated intakes for eight food groups, underestimated intakes for one food group, and showed no statistically significant differences in intakes for four food groups.

**Table 5:** Mean values with standard deviations (SD) for food frequency questionnaire (FFQ) and 24-hour dietary recall (24HR) assessment of daily food group intake, and FFQ as percentage of 24HR in 96 pregnant women

	Mean FFQ ± SD	Mean 24HR ± SD	FFQ % of 24HR
Grains, white roots, and tubers, g	351 ± 200	213 ± 112	165*
Potatoes, unprocessed, g	58.1 ± 55.7	36.8 ± 42.3	158*
Potatoes, processed, g	23.8 ± 36.1	23.4 ± 43.4	102
Vegetables, coarse, g	64.2 ± 57.2	57.7 ± 63.9	111
Vegetables, other, g	76.8 ± 82.0	55.8 ± 56.7	138*
Fruits and berries, g	177 ± 132	133 ± 95.1	133*
Legumes, g	33.5 ± 71.0	27.5 ± 50.2	122
Nuts and seeds, g	5.4 ± 7.8	4.0 ± 7.1	135*
Meat, g	133 ± 99.7	134 ± 79.7	99
Fish and seafood, g	35.6 ± 29.7	23.1 ± 29.1	154*
Eggs, g	17.6 ± 19.3	12.0 ± 22.4	147*
Milk and milk products, g	419 ± 273	368 ± 197	114*
Discretionary foods^a^, g	251 ± 224	308 ± 273	82*

Paired Wilcoxon signed rank test

^a^ Foods such as sweets and desserts, sugary beverages, and processed snacks

* *p* < 0.05

Spearman correlation coefficients for food group intakes assessed using the FFQ and the 24HRs are presented in Table 6. Without energy adjustment, all food groups had acceptable (*r* ≥ 0.3) to very good (*r* ≥ 0.7) deattenuated correlations. Eleven of the 13 food groups had acceptable to very good correlations following energy adjustment, although the distribution within these ranges changed. Two food groups showed small increases in correlation after energy adjustment, seven food groups showed decreases, three food groups maintained the same correlation, and one food group did not remain statistically significant. Notable changes were obtained for legumes, and milk and milk products, which both decreased from very good to good, unprocessed potatoes, which decreased from acceptable to weak, and processed potatoes, which did not remain statistically significant following energy adjustment.

**Table 6:** Spearman correlation coefficients comparing daily food group intake estimates from food frequency questionnaires and 24-hour dietary recalls in 96 pregnant women

Food group	Crude	Deattenuation factor	Deattenuated	Energy adjusted and deattenuated
Grains, white roots, and tubers	0.39*	1.24	0.49*	0.49*
Potatoes, unprocessed	0.26*	1.42	0.38*	0.29*
Potatoes, processed	0.32*	1.31	0.41*	0.17
Vegetables, coarse	0.38*	1.34	0.51*	0.51*
Vegetables, other	0.36*	1.40	0.51*	0.51*
Fruits and berries	0.54*	1.21	0.65*	0.68*
Legumes	0.66*	1.22	0.80*	0.55*
Nuts and seeds	0.32*	1.34	0.42*	0.35*
Meat	0.48*	1.28	0.62*	0.68*
Fish and seafood	0.21*	1.48	0.32*	0.30*
Eggs	0.51*	1.23	0.63*	0.54*
Milk and milk products	0.62*	1.14	0.71*	0.58*
Discretionary foods^a^	0.68*	1.11	0.75*	0.70*

A weak correlation was defined as *r* < 0.3, an acceptable correlation as 0.3 ≤ *r* < 0.5, a good correlation as 0.5 ≤ *r* < 0.7, and a very good correlation as *r* ≥ 0.7

^a^ Foods such as sweets and desserts, sugary beverages, and processed snacks

^*^ *p* < 0.05

As with nutrients, deattenuated values consistently showed stronger correlations than crude values. The mean deattenuated correlation was 0.55, while the mean energy adjusted and deattenuated correlation was 0.49.

Table 7 presents the cross-classification of reported food group intakes into quintiles, comparing estimates from the FFQ with the mean of the 24HRs. Without energy adjustment, most FFQ estimates were correctly classified, with rates ranging from 58% for eggs to 82% for legumes. Following energy adjustment, three food groups showed increases in correct classification, nine food groups showed decreases, and one food group did not change. Notable changes were obtained for processed potatoes, milk and milk products, and legumes, which decreased from 76% to 55%, 78% to 67%, and 82% to 73% respectively, while eggs increased from 58% to 65%. The mean proportion of correctly classified FFQ estimates across all food groups was 71% without energy adjustment, while the mean energy adjusted correct classification was 66%. Gross misclassification rates without energy adjustment ranged from 1% (legumes, eggs, milk and milk products) to 5% (unprocessed potatoes, nuts and seeds, fish and seafood). The mean misclassification rate was 3% regardless of energy adjustment.

**Table 7:** Cross-classification of reported food group intakes into quintiles, comparing estimates from the food frequency questionnaire with the mean of the 24-hour dietary recalls

Food group	Crude		Energy Adjusted	
	Correct classification (%)^a^	Gross misclassification (%)^b^	Correct classification (%)^a^	Gross misclassification (%)^b^
Grains, white roots, and tubers	65	3	67	1
Potatoes, unprocessed	64	5	58	6
Potatoes, processed	76	3	55	5
Vegetables, coarse	67	2	67	3
Vegetables, other	61	2	58	1
Fruits and berries	76	2	74	2
Legumes	82	1	73	2
Nuts and seeds	71	5	67	5
Meat	71	3	73	1
Fish and seafood	69	5	57	7
Eggs	58	1	65	2
Milk and milk products	78	1	67	0
Discretionary foods^c^	81	2	80	3

^a^ Same or adjacent quintile

^b^ Extreme quintiles

^c^ Foods such as sweets and desserts, sugary beverages, and processed snacks

The Bland-Altman plots revealed that two food groups (processed potatoes and discretionary foods) had a statistically significant negative slope indicating a slight systematic trend towards increased underestimation by the FFQ compared to the 24HR with increased intake (Supplementary Fig. 2). Five food groups (fruits and berries, legumes, milk and milk products, vegetables, grains, white roots and tubers) displayed patterns with increased overestimation by the FFQ compared to the 24HR with increasing intake. The remaining six food groups (eggs, fish and seafood, meat, nuts and seeds, unprocessed potatoes, and coarse vegetables) displayed patterns with no systematic differences between the two assessment methods. All food groups had a relatively wide LoA with a small proportion of participants falling outside them.

## Discussion

This study evaluated the relative validity of an FFQ designed for pregnant women in NorthPop by comparing it with repeated 24HRs. The results showed acceptable to very good correlations for most nutrients and food groups, with mean deattenuated correlation coefficients of 0.45 for nutrients and 0.55 for food groups. The FFQ showed good ability to correctly classify participants according to their intake, with mean correct classification rates of 66% for nutrients regardless of energy adjustment, 71% for food groups without energy adjustment, and 66% for food groups following energy adjustment.

The only statistically significant difference between the validation study participants and the general NorthPop participants was observed regarding parity, with fewer women in the validation study having two or more previous pregnancies (8.3% vs. 15.9%). This may affect the generalizability of the findings, as parity status has been associated with variations in dietary patterns [32]. To our knowledge, no studies have shown differences in reporting accuracy based on parity status. Overall, the validation study sample was largely representative of the NorthPop cohort.

The comparison of nutrient mean intakes indicated that the FFQ tended to overestimate the intake of water-soluble nutrients, while underestimating fat-soluble nutrients. Similar patterns of over- and underestimation of dietary intake are commonly observed in FFQ validation studies, as shown in research on pregnant women in Finland [33] and the United Kingdom [34]. These results may be biased by social desirability, whereby participants modify their responses to align with perceived dietary norms [35]. Both underestimation and overestimation in the FFQ compared to the 24HR could also be attributable to the portion sizes used in calculating FFQ intakes. The weight table employed is the only validated source of standard Swedish portion sizes, and its data from 2001 is over two decades old [20]. If average portion sizes have either increased or decreased since 2001, this could contribute to the differences observed. However, discrepancies such as over- and underestimations have limited impact on validity, as the primary purpose of FFQs is to rank individuals according to their relative intake rather than to measure absolute consumption.

The FFQ showed acceptable to very good correlations with most nutrients, both with and without energy adjustment. The mean deattenuated correlation coefficient was 0.45 (0.15 to 0.73) without energy adjustment and 0.45 (0.11 to 0.84) with energy adjustment. These values are similar to those in FFQ validation studies on pregnant women using 24HRs as reference method included in systematic reviews from 2023 and 2009 [12, 29]. For example, studies reported deattenuated energy adjusted mean values of 0.36 (0.11 to 0.63) in China [36] and 0.45 (0.10 to 0.92) in Brazil [37], while a study in the USA reported a deattenuated mean value of 0.50 (0.27 to 0.70) for Caucasian women in the third trimester [38]. Iodine showed a statistically non-significant correlation after energy adjustment, while thiamine showed a non-significant correlation regardless of energy adjustment. The non-significant iodine correlation is concerning given iodine’s importance for fetal neurodevelopment [39], as it may limit the FFQ’s utility for studying iodine-related outcomes. However, food composition data were missing for 206 of the 1261 food items reported in the 24HRs, including commonly consumed foods. These missing values may have systematically underestimated iodine intake in the 24HRs, introducing errors that attenuated the observed correlation. Regarding thiamine, while the 24HRs assigned food item specific thiamine values, several key FFQ items aggregated foods with widely varying thiamine content. For example, the FFQ item for pork products (“Pork: sausage, bacon, ground pork etc.“) encompasses foods ranging from thiamine-rich pork tenderloin to processed sausages with low thiamine content. Similarly, the FFQ items for cereals cover varieties with substantially different thiamine content depending on fortification. Because all participants reporting a given FFQ item are assigned a single thiamine value, individual variation in food choices within these categories is not captured, which may have contributed to the non-significant correlations.

Cross-classification analyses provided further evidence for the FFQ’s ability to rank individuals by nutrient intake. Without energy adjustment, the mean proportion of correctly classified FFQ estimates was 66% across all nutrients. The mean proportion remained stable following energy adjustment. However, individual nutrient estimates shifted, for example fiber (77% to 84%) and niacin equivalents (55% to 66%) improved as energy adjustment removed the effect of total eating volume and isolated the dietary composition signal [13, 27]. In contrast, calcium decreased from 76% to 65%, likely because a substantial proportion of the variation in calcium intake is shared with total energy intake.

Notably, iodine showed improved cross-classification following energy adjustment (53% to 60%) despite having a non-significant energy adjusted correlation. Coupled with the reasonable similarity in mean intake between the FFQ and 24HRs at the group level (where the 20% difference is likely partly explained by the missing 24HR iodine composition data), this suggests the FFQ retains some ability to distinguish between higher and lower iodine consumers even when the linear association is attenuated. Our overall correct classification rates are similar to studies in Norway and Finland, but our misclassification rates (3%) are lower than their 5–8% [33, 40].

The Bland-Altman analysis showed that the LoA were relatively wide across nutrients. This is a common pattern in validation studies of dietary assessment methods [41–43], showing the FFQ has limitations for individual-level use. The systematic trends observed likely reflect social desirability bias and inaccuracies in standard portion size estimations. For nutrients with these trends, the regression line crossed the mean line near the recommended intake according to Nordic nutrition recommendations [44], suggesting that the FFQ is most accurate for capturing intakes within recommended ranges. Outliers in the Bland-Altman analyses came from two sources: reporting of unrealistic intake levels for specific nutrient-rich foods in the FFQ while reporting normal ranges in the 24HRs, or high intake levels reported on days captured by the 24HRs that the FFQ failed to capture.

A comparison of the food group mean intakes indicated that the FFQ tended to overestimate the intake of most food groups, while underestimating discretionary foods (foods such as sweets and desserts, sugary beverages, and processed snacks). This pattern, likely due to social desirability bias, mirrors nutrient reporting trends [35]. As with nutrient intake, these discrepancies have limited impact on the FFQ’s primary function of ranking individuals’ relative intake rather than measuring absolute consumption. All food groups showed acceptable to very good deattenuated correlations before energy adjustment, with a mean of 0.55. After energy adjustment, the correlation of processed potatoes did not remain statistically significant, unprocessed potatoes decreased from acceptable to weak, but the other food groups maintained acceptable to very good correlations, with a mean correlation of 0.49. These values are comparable to those commonly reported in FFQ validation studies on pregnant women, such as a Chinese study that reported a deattenuated energy adjusted mean value of 0.45 (0.35 to 0.56), for food groups [36].

Cross-classification analysis showed that the FFQ effectively distinguished between high and low consumers of food groups. Without energy adjustment, 71% of participants were correctly classified. Following energy adjustment, mean correct classification decreased to 66%, suggesting that some of the agreement was driven by differences in absolute intake rather than composition. For most food groups, the direction of change following energy adjustment was consistent across both cross-classification and correlation analyses. These results mirror our nutrient findings, with the proportion of correctly classified food group intakes comparable to those reported in other FFQ validation studies including the ones in Norway and Finland [33, 40], while our gross misclassification rate was lower. Similar to nutrients, the Bland-Altman analyses for food groups showed relatively wide LoA, with few participants falling outside them. The systematic trends observed are common in validation studies of dietary assessment methods [36, 41].

Strengths of this study include verifying nutrient data availability for all food items and the high completion rate. The scheduling of three 24HRs on nonconsecutive days capturing both weekdays and weekends enhanced the representativeness by capturing potentially different dietary behaviors, while avoiding the interdependence of consecutive days [13]. Both the 24HRs and the FFQ were administered with precise timing, improving validation accuracy. Using a portion guide with photographs in the 24HRs facilitated portion size estimation [45]. Additional strengths include the use of deattenuated correlation coefficients, which showed stronger relationships by accounting for day-to-day variation in the 24HR data. This approach minimizes the attenuation of correlation coefficients [28] and is recommended as standard practice in FFQ validation studies [12]. The use of energy adjustment in correlation analyses is another strength, better reflecting intake proportions more relevant for diet-disease relationships than absolute intake [13]. Energy adjustment improved correlations for some nutrients and food groups while decreasing them for others, consistent with other validation studies [46–48]. This highlights the complex relationship between absolute and energy adjusted intake in dietary assessment. Using multiple statistical approaches (correlation analysis, cross-classification, and Bland-Altman plots) provided a comprehensive assessment of the FFQ validity, with each method offering distinct insights and aligning with recommendations for thorough validation [12, 15, 17].

Limitations of this study include the following: Although dietary supplement use was assessed in a separate questionnaire during pregnancy, this was not included in the present validation study. The supplement questions used a binary yes/no format, without capturing dose or frequency, making validation against 24-hour recalls unfeasible. This is a limitation, given that supplementation is common during pregnancy and can substantially contribute to total nutrient intakes. Nevertheless, as the purpose of the FFQ was to assess intake of foods and food derived nutrient intake, the validity of the FFQ remains supported by the present study. The sample size, though sufficient, is relatively small [13, 15]. Additionally, no correction for day-to-day variation was applied to mean intake, cross-classification, or Bland-Altman plots, making these results crude estimates compared to the deattenuated correlations. All reported energy intakes fell within the predefined plausible range (500–5000 kcal/day). Consequently, no exclusions were made due to implausible energy intake. As these cutoffs are relatively wide, unreliable dietary reports may still be included in our analysis. However, this inclusive approach ensures our results reflect the full range of reporting patterns in the study population. Another limitation was that we did not have available biomarker data. Incorporating biomarkers as an additional reference method would be valuable, especially for nutrients with weaker correlations [13]. Finally, the reproducibility of the FFQ was not examined by repeating its administration, a comparison that could have complemented the relative validity reported here. Conducting such a comparison during pregnancy can be challenging because dietary intake can shift as gestation advances [49]. Furthermore, the time available before delivery is limited, resulting in a relatively narrow interval for data collection. In the present study, the FFQ was completed on a single occasion at 35 weeks of gestation, and reproducibility was not part of the study design. Nonetheless, such an assessment could have been achieved by administering the FFQ twice, as in other pregnancy FFQ validations employing short retest intervals [33, 36]. While the reproducibility of the FFQ remains to be confirmed in future studies, this does not detract from the relative validity reported in the present study.

Both FFQs and 24HRs have inherent advantages and weaknesses. FFQs assess usual intake over time and are less burdensome, while 24HRs provide detailed information [14]. FFQs often overestimate nutrient-dense foods and underestimate energy-dense foods due to social desirability bias [35]. Furthermore, FFQs can be limited by fixed food lists that may not capture individual dietary habits completely, although the inclusion of open-ended questions helps mitigate this limitation. On the other hand, 24HRs are subject to day-to-day variation and may miss food consumed infrequently, potentially affecting results. A limitation of using 24HRs as the reference method for FFQ validation is that both methods involve subjective, retrospective data collection, and thereby share several sources of error, including reliance on memory, estimation of portion sizes, and social desirability bias. Consequently, correlated measurement errors between the two methods may inflate agreement compared with what would be observed using reference methods with more independent errors, such as biomarkers or weighed diet records [13]. Although three 24HRs are adequate for validation [15], they cannot fully capture infrequent consumption patterns [13]. The 24HR method also depends on interviewer skill [35]. Portion size estimation is challenging in both methods, but guides and portion photographs improve accuracy [45].

The FFQ in our study did not specify the exact time-period during pregnancy for which dietary intake is assessed. This may have led participants to recall dietary intake from different time periods between the 24HRs and the FFQs. Combined with dietary changes across trimesters [49], this may have introduced variability that attenuated the observed correlations. However, our results show that the FFQ provides valid measurements for the period of 30–33 weeks of gestation, which is approximately two to five weeks prior to the FFQ administration.

Comparing validation studies is challenging due to differences in correlation methods (Spearman/Pearson), energy adjustment techniques, deattenuation application, reporting of crude/energy adjusted/deattenuated values, and reference methods. Future FFQ validation studies would benefit from addressing these inconsistencies. Using Spearman rank correlation coefficients exclusively may be preferable, as FFQs rank individuals rather than assess absolute intake, and nutrient/food group intake variables typically do not follow normal distributions [13]. Establishing consensus on reporting crude and deattenuated values, with or without energy adjustment, would facilitate comparisons between studies. Standardizing quantiles (tertiles, quartiles, or quintiles) for cross-classification and thresholds for correlation strength would also improve methodological consistency and result interpretability. In FFQ research, it is important to manage outliers for individual nutrients and food groups, not just for energy intake. Our analysis found extreme outliers for specific nutrients and food groups that could influence results, even when energy intake appeared reasonable. This suggests a need for a more comprehensive approach to outlier management. Updated data for Swedish standard portion sizes is also needed.

## Conclusion

The FFQ evaluated in this study is a valid tool for assessing dietary intake at the group level among pregnant women in the NorthPop cohort. The results support the use of this FFQ to study the relationships between dietary factors and disease during pregnancy and beyond for mother and offspring.

## Supplementary Information

Additional file 1: Table S1: The STROBE-nut checklist. Table S2: Food group classification. Figure S1: Bland-Altman plots with regression lines comparing energy adjusted nutrient intakes assessed by FFQ and the 24HRs. Figure S2: Bland-Altman plots with regression lines comparing energy adjusted intakes of food groups assessed by FFQ and the 24HRs.

## Data Availability

Individual participant data are not publicly available under the General Data Protection Regulation (GDPR). Anonymized data can be accessed following a reasonable request, necessary ethical approvals, and approval from the NorthPop steering group at Umeå University and Region Västerbotten, Sweden.

## Abbreviations

24HR: 24-hour dietary recall
BMI: Body mass index
d: Day
FFQ: Food frequency questionnaire
LoA: Limits of agreement
NorthPop: The NorthPop Birth Cohort Study
SD: Standard deviation
